# Knowledge, attitudes and uptake related to influenza vaccine among healthcare workers during the 2018–2019 influenza season in Tunisia

**DOI:** 10.1186/s12889-021-10970-y

**Published:** 2021-05-13

**Authors:** Ines Cherif, Ghassen Kharroubi, Leila Bouabid, Adel Gharbi, Aicha Boukthir, Nissaf Ben Alaya, Afif Ben Salah, Jihene Bettaieb

**Affiliations:** 1 Laboratory of Medical Epidemiology, Pasteur Institute of Tunis, 13, Place Pasteur, B.P.74, Belvédère, 1002 Tunis, Tunisia; 2 Laboratory of Transmission, Control and Immunobiology of Infections (LR11IPT02), Pasteur Institute of Tunis, 13, Place Pasteur, B.P.74, Belvédère, 1002 Tunis, Tunisia; 3 National Observatory of New and Emerging Diseases, 5-7, Khartoum Street, Diplomat, 13th floor, Le Belvédère, 1002 Tunis, Tunisia; 4 Arabian Gulf University, Road 2904 Building 293, Manama, 329 Bahrain

**Keywords:** Influenza, Vaccination, Attitude, Knowledge, Health personnel, Tunisia

## Abstract

**Background:**

The influenza vaccine (IV) is considered the most effective strategy to prevent seasonal influenza infection and annual vaccination of healthcare workers (HCWs) is recommended by the World Health Organization given their high mixing with patients. We assessed IV uptake among HCWs in the 2018–2019 season and explored their knowledge and attitudes regarding influenza immunization.

**Methods:**

A cross-sectional study was conducted in 150 representative Tunisian health facilities from March to May 2019. We recruited 1231 HCWs with direct patient contact using self-weighted multistage sampling. Univariate and multivariate logistic regression analyses permitted to assess the factors associated with IV uptake in the 2018–2019 influenza season.

**Results:**

Among 1231 health professionals enrolled in this study, less than half (36.6, 95% confidence interval [CI]: 33.9–39.4) received the IV at least once in their lives and only 15.3% (CI: 13.3–17.4) were vaccinated against influenza in the 2018–2019 influenza season. High confidence regarding IV efficacy, belief about the mandatory character of influenza vaccination for HCWs, and IV uptake in the 4 years preceding the 2018–2019 influenza season were independently associated with higher IV uptake by multivariate analysis. However, participants with high educational level were less likely to receive the IV than those with the lowest educational level.

**Conclusions:**

Our study revealed a low vaccination rate among Tunisian HCWs confirming the importance of tailored education programs targeting this population.

**Supplementary Information:**

The online version contains supplementary material available at 10.1186/s12889-021-10970-y.

## Background

Influenza is an acute respiratory infection that is highly contagious and considered one of the most challenging public health problems worldwide [1, 2]. It may range from mild to severe illness causing hospitalizations and deaths mainly among high-risk groups. Globally, an estimated 1 billion cases of influenza occur each year, with 3 to 5 million cases of severe illness and 290,000 to 650,000 influenza-related deaths [1, 3]. In Tunisia, an average of 130,000 to 272,000 cases of influenza-like illness are recorded each year at influenza sentinel surveillance sites, representing 6.5 to 12.9% of all outpatient visits [4]. In addition, 119 cases of severe acute respiratory illness due to influenza and 48 influenza-related deaths were reported from the beginning of the 2017–2018 influenza season to the end of January 2018 [5].

Given their close and regular contact with ill patients, healthcare workers (HCWs) are at a high risk of developing influenza and may transmit the disease to their patients. As such, HCW infections may result in nosocomial outbreaks, with an increased risk of mortality among immunocompromised hospitalized patients [6]. Influenza infection among health professionals was also associated with a high economic burden mainly related to absenteeism [7].

Influenza vaccines (IVs) have been available since 1945 and remain the most effective tool to prevent influenza infection and its complications [1, 8]. HCWs immunization is a cost-effective method proven to reduce influenza-related deaths among high-risk patients [9, 10]. Furthermore, vaccination of HCWs can protect patients who cannot receive the vaccine or those who respond poorly to vaccination [11].

Given the aforementioned reasons, the World Health Organization (WHO) and the U.S. Advisory Committee on Immunization Practices (ACIP) have recommended annual vaccination of HCWs [1, 11]. Despite these recommendations and the efficacy of HCW influenza vaccination in improving self and patients’ safety, vaccine coverage among healthcare professionals remains low mainly in developing countries [12, 13]. Although the IV is provided free of charge to health professionals in Tunisia, the estimated vaccination uptake proportion among HCWs remains low [14]. However, this indicator has never been accurately assessed at the national level and its determining factors are not fully understood.

We performed this nationwide study to measure the vaccine uptake and to understand the knowledge and attitudes related to influenza vaccination among HCWs, in order to propose evidence-based strategies to address these gaps in Tunisia.

## Methods

### Study design and population

A cross-sectional study was conducted in Tunisian primary health care centers, regional and district hospitals between March and May 2019.

HCWs were recruited from 150 health facilities (66 in northern Tunisia, 62 in the center and 22 in southern Tunisia). The study included all HCWs with direct patient contact at participating health facilities. Direct patient contact was defined as in-person, face-to-face contact between a healthcare provider and a patient, including patient registration, education, counseling, treatment, or any other aspect of patient health care.

### Sampling process

**Fig. 1:**
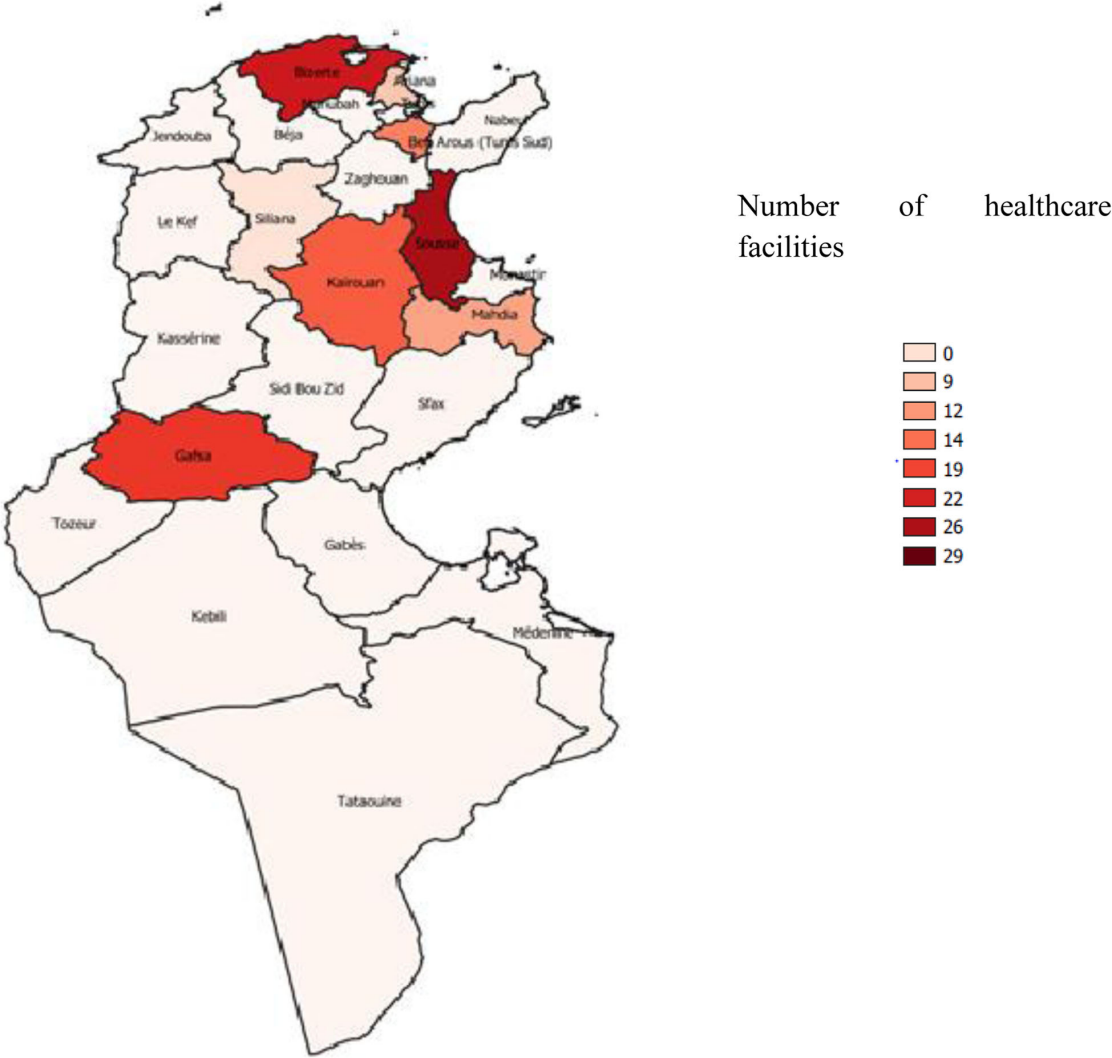
Distribution of selected health facilities by governorates

HCWs were recruited according to self-weighted multistage sampling. Stage 1 used stratified sampling according to Tunisian regions (north, center, and south). Eight of the 24 Tunisian governorates were selected randomly: four in the north (Ariana, Ben Arous, Bizerte, Siliana), three in the center (Kairouan, Mahdia, Sousse), and one in the south (Gafsa) (Fig. 1).

Stages 2 and 3 utilized stratified sampling according to governorates and cluster sampling of healthcare facilities, respectively.

The calculated sample size was distributed according to the distribution of Tunisian HCWs between the three Tunisian regions, to the weight of each governorate in the corresponding region and to the distribution of physicians and other HCWs in each of the selected governorates.

### Sample size

The sample size was estimated for a 2.5 design effect and a 20% non-response rate, using Slovin’s formula:

$$ n=\mathrm{N}/\left(1+N{\left(\mathit{\exp}\right)}^2\right), $$

N: Total population of the target group; exp.: Desired precision.

Assuming a precision of 0.05 and expected target population sizes in primary health care centers, regional and district hospitals of between 10,000 and 100,000, the estimated sample size was ≃1200 HCWs.

### Data collection

Data were collected using a face to face questionnaire composed of two sections (Additional file 1: Appendix A.1). The first section focused on HCW uptake of IV and their attitudes and knowledge regarding influenza and IV. Open-ended questions were used to assess reasons for vaccine acceptance or refusal and knowledge of priority target groups for vaccination. General statements related to knowledge and attitudes included items about influenza dangerousness and contagiousness and about IV efficacy and safety. These statements were assessed using a 5-point Likert scale (1: strongly disagree, 2: disagree, 3: neither agree nor disagree /I don’t know, 4: agree, 5: strongly agree). Respondents were also asked to rate their confidence regarding the ability of the IV to prevent influenza among HCWs on a scale of 1 to 5 (from 1: not at all to 5: very much). The second section contained questions about HCW sociodemographic characteristics.

We conducted a pilot study to train investigators and assess the clarity and comprehensibility of the survey.

The directors of the selected healthcare facilities were informed beforehand of the date of the investigators’ visit. Trained staff approached HCWs in their workplace to solicit their interest and consent. Those who agreed to participate were asked to respond anonymously to the survey questionnaire.

Agreements and approvals from the health authorities were sought prior to data collection to meet regulatory requirements and ensure maximal proportion’s response.

### Statistical analysis

HCWs were categorized as physicians, paramedics (nurses, assistant nurses, midwives and healthcare technicians), or other HCWs (healthcare assistants, administrative staff, psychologists, and pharmacists). To facilitate the interpretation of study results, Likert scale responses to general statements related to knowledge and attitudes were dichotomized by grouping “I don’t know” responses with “neutral,” “disagree,” and “strongly disagree,” as “other responses” and “strongly agree” responses with “agree.” Likewise, we dichotomized responses to the question related to confidence toward IV efficacy as follows: answers rated from 1 to 3 were assigned to low confidence while those from 4 to 5 were assigned to high confidence. “I don’t know” answers were not included in the univariate analysis except for knowledge and attitude questions that were assessed using a 5-point Likert scale.

χ^2^ tests for univariate analysis and logistic regression for multivariate analysis were used to identify factors significantly associated with IV uptake in the 2018–2019 influenza season among Tunisian HCWs. Variables that had a *p* value less than or equal to 0.2 in univariate analysis were included in multivariate analysis.

Data were entered and analyzed using Epi Info version 7.2.2.6 (Developed by Centers for Disease Control and Prevention, U. S (US CDC)).

## Results

Overall, 1359 HCWs were approached to participate in the survey. Among them, 1264 accepted to respond to the questionnaire (Response rate = 93%). Among the collected questionnaires, 33 were removed owing to high percentages of missing responses and not meeting the inclusion criteria. The remaining 1231 questionnaires (97%) were eligible for analysis. Most of the participants were women (80.0%). Their mean age was 44.5 ± 9.3 years, ranging from 22 to 64 years. More details on the participants’ profession and sociodemographic characteristics are presented in Table 1.

**Table 1:** Sociodemographic characteristics of a sample of Tunisian health care workers in 2019

Characteristics	n (%)
Gender (*n* = 1227)	
Male	245 (20.0)
Female	982 (80.0)
Age (years) (*n* = 1135)	
[20–30[	55 (4.8)
[30–40[	328 (28.9)
[40–50[	339 (29.9)
≥ 50	413 (36.4)
Educational level (*n* = 1231)	
Primary school or less	65 (5.3)
Secondary school/vocational training	521 (42.3)
University degree	645 (52.4)
Occupation (*n* = 1231)	
Physicians	182 (14.8)
Paramedics	855 (69.5)
Other HCWs^a^	194 (15.7)
Average number of patients seen per day (*n* = 1223)	
≤ 10	103 (8.4)
11–30	385 (31.5)
> 30	735 (60.1)
Professional Experience (years) (*n* = 1225)	
< 5	80 (6.5)
[5–15]	457 (37.3)
[15–25]	339 (27.7)
≥ 25	349 (28.5)
Health facility type (*n* = 1231)	
Primary health care centers	859 (69.8)
District hospitals	256 (20.8)
Regional hospitals	116 (9.4)

^a^Healthcare workers

Among respondents, 36.6% [33.9–39.4] reported receiving the IV at least once in their lives, while 27.6% [25.1–30.2] had received the IV at least once during the 4 years preceding the 2018–2019 influenza season and 15.3% [13.3–17.4] were vaccinated during the 2018–2019 influenza season. Most of participants 75.9% [73.4–78.3] reported that the IV is available for free to HCWs at their workplace. More than half of the respondents (53.8% [50.9–56.6]) declared their unwillingness to receive the IV even if recommended to HCWs and provided for free at their workplace and 65.3% [62.6–68.0] reported low confidence regarding vaccine efficacy in preventing influenza among healthcare personnel while 79.1% [76.7–81.3] declared their willingness to recommend or prescribe the IV to patients if available.

According to participants, the main three reasons leading to vaccine acceptance were: self-protection from influenza (73.8% [71.2–76.2]), family and colleagues’ protection (49.2% [46.4–52.1]) and protection of patients (28.2% [25.7–30.9]). Fear of the vaccine side effects (48.0% [45.2–50.9]), not feeling at risk of influenza (31.8% [29.2–34.5]) and doubt about vaccine efficacy (31.6% [28.9–34.3]) were the most frequent cited reasons leading to IV refusal.

Most participants believed that influenza may result in severe illness or death (80.0% [77.6–82.2]) and that they could transmit it to their family members (91.9% [90.3–93.4])). More than half agreed that vaccinating HCWs could reduce work absenteeism, severe illness and deaths among patients (54.9% [52.1–57.7] and 52.7% [49.8–55.5] respectively). However, almost half (48.5% [45.6–51.3]) of the participants believed that the IV could cause influenza. In addition, 29.2% [26.7–31.8] of respondents were aware that HCWs are a target group for influenza vaccination and almost three-quarters (74.5% [72.0–76.9]) knew that the IV was recommended annually for HCWs.

Univariate analysis showed that HCWs belonging to primary healthcare centers were 2.4 times more likely to be vaccinated against influenza in 2018–2019 than those working in regional and district hospitals (*p* < 0.001). We also observed a significant association between the participants’ educational level and vaccination status (*p* = 0.005), with the highest percentage of vaccine uptake among those with the lowest educational level. Besides that, the vaccination rate increased with age (*p* < 0.001), professional experience (p < 0.001) and with the average number of patients seen per day (*p* = 0.038). Gender and type of occupation were not significantly associated with 2018–2019 vaccination uptake (Additional file 1: Appendix A.2).

HCWs who highly trust the vaccine efficacy in preventing influenza, and those who believed that HCW vaccination against influenza could reduce severe illness and deaths in patients, as well as those who support a mandatory IV among HCWs were more likely to be vaccinated than their counterparts (OR = 3.5, *p* < 0.001; OR = 1.7, *p* = 0.001 and OR = 2.8, *p* < 0.001 respectively).

Respondents who were aware that annual influenza vaccination is recommended for HCW had significantly higher vaccination rates than other respondents (*p* < 0.001) and vaccine uptake in 2018–2019 was higher among those who had received the vaccine at least once in the four preceding seasons (42.2% vs 5.0%) (Table 2).

**Table 2:** Influenza vaccine uptake in 2018–2019 by healthcare workers’ knowledge attitudes and practices related to influenza vaccine-Tunisia, 2019

Variables	N	IVU^**a**^ in 2018–2019 n (%)	Crude OR [95% CI]^**b**^	***P*** value
Previous influenza vaccine uptake (in the 4 years preceding the 2018–2019 influenza season)				< 0.001
Yes	339	143 (42.2)	13.9 [9.6–20.2]	
No	884	44 (5.0)	1	
Availability of influenza vaccine for free at the health facility				< 0.001
Yes	932	168 (18.0)	2.9 [1.7–4.9]	
No	245	17 (6.9)	1	
HCWs^c^ are a target group for influenza vaccination				< 0.001
Yes	359	80 (22.3)	2.0 [1.5–2.8]	
No	869	107 (12.3)	1	
Influenza vaccine is indicated annually for HCWs				< 0.001
Agree	909	167 (18.4)	3.1 [1.9–4.9]	
Others	311	21 (6.8)	1	
Confidence regarding influenza vaccine efficacy in preventing influenza among HCWs				< 0.001
High confidence	421	111 (26.4)	3.5 [2.5–4.8]	
Low confidence	791	74 (9.4)	1	
Willingness to vaccinate or recommend the influenza vaccine to patients				0.001
Yes	969	166 (17.1)	3.3 [1.6–6.8]	
No	135	8 (5.9)	1	
Influenza may result in severe illness or death				0.196
Agree	980	156 (15.9)	1.3 [0.9–1.9]	
Others	246	31 (12.6)	1	
HCWs can transmit influenza to their family members				0.018
Agree	1125	180 (16.0)	2.5 [1.1–5.5]	
Others	99	7 (7.1)	1	
Vaccination of HCWs can reduce influenza including severe illness and/or deaths in patients				0.001
Agree	647	119 (18.4)	1.7 [1.2–2.3]	
Others	579	68 (11.7)	1	
Influenza vaccine in this country should be mandatory for HCWs				< 0.001
Agree	536	123 (22.9)	2.8 [2.1–3.9]	
Others	687	65 (9.5)	1	
The influenza vaccine can cause a person to get sick with influenza				0.924
Agree	594	90 (15.2)	1.0 [0.7–1.4]	
Others	632	97 (15.3)	1	
Vaccinating HCWs may reduce work absenteeism				0.001
Agree	672	123 (18.3)	1.7 [1.2–2.4]	
Others	550	63 (11.5)	1	

^a^Influenza vaccine uptake

^b^Odds ratio [95% Confidence Interval]

^c^Healthcare workers

The independent factors associated with IV uptake in 2018–2019 among Tunisian HCWs are summarized in Table 3. Variables included in the initial multivariate model were: Educational level, age, average number of patients seen per day, health facility type, previous IV uptake, confidence regarding vaccine efficacy in preventing influenza, willingness to vaccinate or recommend IV to patients, availability of IV for free, believing that HCWs are a target for influenza vaccination, that IV is indicated annually for HCWs, that influenza may result in severe illness or deaths, that HCWs can transmit influenza to their family members, that IV should be mandatory for HCWs and that it can reduce work absenteeism and severe illness among patients.

**Table 3:** Predictors of influenza vaccine uptake among Tunisian healthcare workers in 2018–2019 influenza season

Variables	OR_**a**_^**a**^	[CI _**95%**_]^**b**^
Educational level		
Primary school or less	1	
Secondary school/Vocational training	0.29	[0.11–0.79]
University degree	0.22	[0.08–0.58]
Previous influenza vaccine uptake (in the 4 years preceding the 2018–2019 influenza season)		
Yes	13.19	[8.37–20.81]
No	1	
Influenza vaccine in this country should be mandatory for healthcare workers		
Agree	1.63	[1.05–2.53]
Others	1	
Confidence regarding vaccine efficacy in preventing influenza		
High confidence	2.07	[1.34–3.17]
Low confidence	1	

^a^adjusted odds ratio

^b^95% Confidence Interval

## Discussion

We assessed IV coverage among Tunisian healthcare professionals working in primary healthcare centers, district and regional hospitals in the 2018–2019 season and described HCW knowledge and attitudes regarding influenza and IV. Fewer than half of respondents (36.6%) have received the vaccine at least once in their lives and only 15.3% were vaccinated in the 2018–2019 influenza season. Moreover, more than half of the participating HCWs reported their unwillingness to receive the IV even if provided for free. The most commonly cited reason for vaccine refusal was fear of vaccine side effects. Multivariate analysis revealed a significant association between IV uptake in 2018–2019 and previous IV receipt, educational level, belief that the IV should be mandatory for HCWs, and confidence regarding IV efficacy in preventing influenza.

Globally, influenza vaccination rates previously reported among HCWs studies have varied [12, 13, 15–25]. The reported vaccine uptake among our respondents for the 2018–2019 season was much lower than those reported in studies conducted in some countries of the Middle East and North Africa (MENA) region, where the IV is provided freely to health professionals. In Saudi Arabia, two studies conducted in similar healthcare settings reported an increase in HCW vaccination rates between the 2012–2013 and 2016–2017 seasons – from 38 to 67.6% [22, 23]. Similarly, a study conducted in a community hospital in Qatar found that more than half of health professionals were vaccinated against influenza in 2011–2012 and exceeded 70% during the 2012–2013 influenza season [26]. In addition, the administrative vaccination coverage of health professionals against influenza in Morocco was estimated 54% in 2016 [27].

Despite international recommendations for annual vaccination of health professionals and the provision of IV free of charge to HCWs in Tunisia, the vaccination rate is low. This might be explained by the lack of appropriate flu awareness campaigns in Tunisian health care facilities. Thus, health authorities should pay more attention to raise awareness of health professionals toward the necessity of influenza immunization through annual educational programs that could be delivered online. Facilitating access to influenza immunization by making time of vaccine delivery more flexible should also be considered [28]. Reminder messages through social networks and media before and during the influenza season may also help to increase vaccine coverage [28].

The most important predictor of IV uptake was the previous vaccination during the previous 4 years. However, we did not observe a significant association between vaccination status and professional category. Surprisingly, HCWs with the lowest educational level were more likely to be vaccinated against influenza in 2018–2019 compared to their counterparts. Our results are in contrast to those reported by Hammour et al. [29], in which HCW vaccine uptake increased with educational level. In our study, the highest vaccine uptake among those with the lowest educational level might be explained by a lower concern about IV side effects than those with higher education level. Vaccine uptake in 2018–2019 was higher among healthcare professionals working in primary care centers than among those working in regional and district hospitals despite their more frequent contact with patients at high risk of complications. This could be explained by higher exposure of general practitioners to the recommendations of the national influenza control program. Therefore, national educational programs should focus mainly on health professionals in contact with vulnerable patients.

Vaccine uptake among HCWs was associated with their willingness to recommend IV to their patients (*p* = 0.001). Our results are consistent with those of Joseph et al. [16] who reported a positive association between IV uptake among French general practitioners and vaccine coverage among patients aged 65 years and above. Other studies, conducted among elderly, adults with chronic diseases and pregnant women, found that recommendations from healthcare providers are one of the leading causes of IV vaccine acceptance and receipt among patients [30–32] and that they are the main source of information about influenza [31]. The low influenza immunization coverage in Tunisia may be partly attributed to low level of confidence regarding vaccine efficacy which is translated in low vaccine acceptance among prescribers. In agreement with Petek et al. [18], we observed an independent positive association between high confidence in vaccine efficacy and IV uptake in the 2018–2019 season (OR: 2.07).

Main barriers to vaccine acceptance for HCWs were fear of IV side effects, low perceived risk of severe influenza disease and doubt about vaccine efficacy. As expected, these reasons corroborate those reported globally [17–19, 21, 23, 25, 33] and support the presence of misconceptions regarding influenza and influenza vaccines among HCWs. Indeed, almost half of our study sample believed that IV could cause a person to develop influenza. These results are in agreement with those from Saudi Arabia, where we found that 48.2% of health professionals believed mistakenly that the IV included live virus and could cause influenza [23]. Although IV containing live viruses does exist, it cannot cause influenza. Indeed, viruses contained in these vaccines are attenuated [34].

Despite the ACIP and WHO recommendations, less than one-third of Tunisian respondents identified HCWs as a target group for influenza immunization. These results are in contrast with previous studies in India and Saudi Arabia, in which the majority of surveyed HCWs were aware that the IV was recommended to HCWs [12, 22, 23]. Similarly, a study conducted during the 2018–2019 influenza season among healthcare providers working in critical care units in Italy found that more than half (64.1%) of participants knew that IV is recommended for HCWs [25].

Our results underscore the urgent need to educate HCWs about IV target groups and the vaccine composition. Tunisian vaccination awareness programs should also include information on the rates of IV side effects in addition to those of severe illnesses and deaths averted due to vaccination to raise HCW confidence regarding influenza immunization. In addition, COVID-19 pandemic might be a great opportunity to promote IV. Indeed, COVID-19 and influenza are both infectious respiratory diseases that have some symptoms and complications in common causing respiratory distress, hospitalization in intensive care units and even deaths mainly among vulnerable persons. HCWs encouraging the use of a vaccine against SARS-COV-2 just released after emergency use authorization, may find it hard to justify their negative attitude regarding flu shot that is already available for decades.

As educational programs alone may not be sufficient to increase vaccine uptake among health professionals, the Healthcare Infection Control Practices Advisory Committee (HICPAC) and the ACIP recommend the requirement of a signed declination from HCWs if they refuse to receive the IV without having any medical contraindications [35].

Mandatory vaccination of Tunisian health workers may be a solution to increase the uptake of IV among HCWs; however, only 42.8% of the present study respondents agreed with this action. Other studies observed higher percentages of mandatory vaccine acceptance among healthcare providers [13, 36].

Although more than half of respondents believed that vaccinating HCWs can reduce severe illness and deaths among patients, fewer than one-third mentioned patient protection as a main reason for vaccine acceptance. Indeed, self and family protection appeared to be more motivating than patient safety. These results are consistent with those of other studies, in which self-protection was identified as the main reason for vaccine acceptance [21, 37].

### Study strengths and limitations

To our knowledge, this is the first study of its kind in Tunisia as well as in North Africa; thus, it provides benchmark data for use by health authorities to tailor and improve local IV strategies. Moreover, assessing IV acceptance among HCWs will help to forecast vaccine supply needs in order to avoid vaccine wastage and its related costs.

Given the COVID-19 pandemic, understanding barriers toward IV uptake among Tunisian HCWs may also help to implement effective awareness campaigns to promote SARS-CoV-2 vaccine.

Despite the fact that we conducted a national study among a randomized sample of Tunisian HCWs, we could not verify the representativeness of our sample since we don’t have an updated information of the sociodemographic characteristics of health professionals working in primary healthcare centers, regional and district hospitals in Tunisia overall. Besides that, there may be a recall bias when checking the vaccination history through a questionnaire. Indeed, the collection of this information was based solely on the respondents’ statements and the unavailability of a vaccination record or any other official document testifying of IV uptake among HCWs prevented us from verifying the accuracy of these statements.

For feasibility reasons, we did not include health professionals from the private sector and those working in university hospitals which indicate that our findings are not generalizable to all Tunisian HCWs.

Future studies focusing on the tertiary care level health professionals and those in the private sector are planned to get a more comprehensive picture.

## Conclusions

Despite recommendations, the vaccination rates were low among Tunisian health professionals. The low vaccination uptake may be related to a lack of confidence regarding IV efficacy and misconceptions about influenza immunization. These findings highlight the need for educational programs to raise HCWs’ awareness of vaccine efficacy and safety. Mandatory vaccination policies in healthcare facilities may also be considered. Findings from the present study can be useful to overcome potential barriers against the uptake of COVID-19 vaccine among HCWs which are identified as a priority group.

## Supplementary Information

**Additional file 1: Appendix A1.** Survey Questionnaire. **Appendix A2.** Influenza vaccine uptake in 2018-2019 according to healthcare workers’ characteristics-Tunisia, 2019.

## Data Availability

The datasets used and/or analyzed during the current study are available from the corresponding author on reasonable request.

## Abbreviations

IV: Influenza vaccine
HCWs: Healthcare workers
WHO: World Health Organization
ACIP: Advisory Committee on Immunization Practices
CDC: Centers for Disease Control and Prevention
HICPAC: Healthcare Infection Control Practices Advisory Committee
