# Supplementary material for: Knowledge, attitudes and uptake related to influenza vaccine among healthcare workers during the 2018–2019 influenza season in Tunisia
Source: BMC Public Health. 2021 May 13;21:907. doi: 10.1186/s12889-021-10970-y (PMC8116062; doi:10.1186/s12889-021-10970-y)
Supplement: Supplementary file 1 — Additional file 1: Appendix A1. Survey Questionnaire. Appendix A2. Influenza vaccine uptake in 2018-2019 according to healthcare workers’ characteristics-Tunisia, 2019. [file 12889_2021_10970_MOESM1_ESM.docx]

**Appendix A.1: Survey Questionnaire**

**Knowledge, Attitudes, and Practice Survey Questionnaire: Health Care Workers**

**Date of interview**: ________________ **Name of interviewer**: _______________________

**Health Facility Name:** ______________ **Health Facility Type**: _____________________

**Location**: ________________________

1. **Questions about history, general knowledge and attitudes about influenza and influenza vaccines**
2. **Have you ever been vaccinated against influenza?**
3. ☐ Yes
4. ☐ No (Skip to Question 4)
5. ☐ I don’t remember (Skip to Question 4)
6. **Did you receive the influenza vaccine during the influenza season (2018-2019)?**
7. ☐ Yes
8. ☐ No
9. ☐ I don’t remember
10. **How many times did you receive influenza vaccine in the past 5 years?**
11. ☐ Never
12. ☐ Once
13. ☐ 2 to 4 times
14. ☐ 5 times
15. ☐ I don’t remember
16. **Is influenza vaccine available for free to health care workers at the health facility where you work?**
17. ☐ Yes
18. ☐ No
19. ☐ I don’t know
20. **If influenza vaccine was recommended for health care workers, and provided for free, will you accept to receive the vaccine?**
21. ☐ Yes
22. ☐ No (Skip to Question 7)
23. ☐ I don’t know
24. **On a scale of 1 to 5, 1 being “not at all” and 5 being “very much”, how confident are you that influenza vaccine can prevent influenza in health care workers?**
25. ☐ 1
26. ☐ 2
27. ☐ 3
28. ☐ 4
29. ☐ 5
30. **What are the main reasons that would make you ACCEPT to take the influenza vaccine for yourself?**

**____________________________________________________________________________________________________________________________________________________________________________________________________________________________________________________________**
☐ I don’t know

1. **What are the main reasons that would make you REFUSE to take the influenza vaccine for yourself?**

**____________________________________________________________________________________________________________________________________________________________________________________________________________________________________________________________**

☐ I don’t know

1. **If influenza vaccine was available for your patients, would you vaccinate your patients or recommend they receive the vaccine?**
2. ☐ Yes
3. ☐ No
4. ☐ I don’t know
5. **Please indicate the main groups of people you would vaccinate or would recommend they receive the vaccine against influenza.**

**____________________________________________________________________________________________________________________________________________________________________________________________________________________________________________________________**

☐ I don’t know

1. **Below are 7 general statements about influenza and influenza vaccine. After reading each statement, please indicate if you agree OR disagree with the statement. If you don’t know, please indicate by checking the corresponding box.**

|  | **Strongly Agree** | **Agree** | **Neutral** | **Disagree** | **Strongly Disagree** | **I don’t know** |
| --- | --- | --- | --- | --- | --- | --- |
| a. Influenza may result in severe illness or death | ☐ | ☐ | ☐ | ☐ | ☐ | ☐ |
| b. Healthcare workers can transmit influenza to their family members. | ☐ | ☐ | ☐ | ☐ | ☐ | ☐ |
| c. Vaccinating healthcare workers may reduce work absenteeism | ☐ | ☐ | ☐ | ☐ | ☐ | ☐ |
| d. Vaccination of healthcare workers can reduce influenza including severe illness and/or deaths in patients | ☐ | ☐ | ☐ | ☐ | ☐ | ☐ |
| e. The influenza vaccine can cause a person to get sick with influenza | ☐ | ☐ | ☐ | ☐ | ☐ | ☐ |
| f. Influenza vaccine in this country should be mandatory for health care workers | ☐ | ☐ | ☐ | ☐ | ☐ | ☐ |
| g. Influenza vaccine is indicated annually for healthcare workers | ☐ | ☐ | ☐ | ☐ | ☐ | ☐ |

1. **General and Demographic Questions**
2. **What is your gender?**
   1. ☐ Male
   2. ☐ Female
3. **What is your age? (years) (or date of birth): …………………………………………………..**
4. **What is the highest level of education you have completed?**
   1. ☐ Primary school or less
   2. ☐ Secondary school
   3. ☐ Vocational training
   4. ☐ University degree
   5. ☐ Other: _______________________
5. **What is your occupation?**
   1. ☐ Medical Doctor
   2. ☐ Dentist
   3. ☐ Nurse
   4. ☐ Assistant Nurse
   5. ☐ Midwife
   6. ☐ healthcare technicians
   7. ☐ Pharmacist
   8. ☐ Healthcare assistant
   9. ☐ Administrative staff
   10. ☐ Other, specify ______________________________
6. **On average, how many patients do you see per day?**
   1. ☐ 0-10
   2. ☐ 11-20
   3. ☐ 21-30
   4. ☐ 31-40
   5. ☐ More than 40
7. **How many years have you worked in health care?**
8. ☐ Less than 1 year
9. ☐ 1 to 4 years
10. ☐ 5 to 9 years
11. ☐ 10 to 14 years
12. ☐ 15 to 19 years
13. ☐ 20 to 24 years
14. ☐ 25 to 29 years
15. ☐ 30+ years
16. **Do you have comments or suggestions regarding influenza vaccine for health care workers?**

**______________________________________________________________________________________________________________________________________________________________________________________________________________________________________________________________________________________________________________________________________________________________________________________________________**

**Thank you for completing this questionnaire**

**Appendix A2: Influenza vaccine uptake in 2018-2019 according to healthcare workers’ characteristics-Tunisia, 2019**

| **Characteristics** | **N** | **IVU^a^ in**  **2018-2019**  **n (%)** | **Crude**  **OR [95% CI]^b^** | **P value** |
| --- | --- | --- | --- | --- |
| Gender |  |  |  | 0.584 |
| Women | 981 | 147 (15.0) | 1 |  |
| Men | 244 | 40 (16.4) | 1.1 [0.8-1.6] |  |
| Age (years) |  |  |  | <0.001 |
| [20-30[ | 55 | 3 (5.5) | 1 |  |
| [30-40[ | 327 | 35(10.7) | 2.1 [0.6-7.0] |  |
| [40-50[ | 339 | 45 (13.3) | 2.6 [0.8-8.8] |  |
| ≥50 | 412 | 93 (22.6) | 5.0 [1.5-16.5] |  |
| Educational level |  |  |  | 0.005 |
| Primary school or less | 65 | 16 (24.6) | 1 |  |
| Secondary school/Vocational training | 520 | 92 (17.7) | 0. 7 [0.4-1.2] |  |
| University degree | 644 | 80 (12.4) | 0.4 [0.2-0.8] |  |
| Occupation |  |  |  | 0.624 |
| Doctor | 181 | 28 (15.5) | 1 |  |
| Paramedics | 854 | 126 (14.8) | 0.9 [0.6-1.5] |  |
| Other hcws | 194 | 34 (17.5) | 1.2 [0.7-2.0] |  |
| Average number of patients seen per day |  |  |  | 0.038 |
| ≤10 | 103 | 7 (6.8) | 1 |  |
| [11-30[ | 385 | 60 (15.6) | 2.5 [1.1-5.7] |  |
| >30 | 733 | 121 (16.5) | 2.7 [1.2-5.9] |  |
| Professional experience (years) |  |  |  | <0.001 |
| <5 | 79 | 6 (7.6) | 1 |  |
| [5 - 15[ | 457 | 51 (11.2) | 1.5 [0.6 -3.7] |  |
| [15 - 25[ | 339 | 52 (15.3) | 2.2 [0.9-5.3] |  |
| ≥25 | 348 | 77 (22.1) | 3.5 [1.4-8.2] |  |
| Health facility type |  |  |  | <0.001 |
| Primary health care centers | 859 | 157 (18.3) | 2.4 [1.6-3.7] |  |
| Regional and district hospitals | 370 | 31 (8.4) | 1 |  |

^a^: Influenza vaccine uptake

^b^: Odds ratio [95% Confidence Interval]
